# Characteristics of spontaneous coagulase-negative staphylococcal spondylodiscitis: a retrospective comparative study versus *Staphylococcus aureus* spondylodiscitis

**DOI:** 10.1186/s12879-017-2783-0

**Published:** 2017-10-13

**Authors:** Julien Lopez, Zuzana Tatar, Anne Tournadre, Marion Couderc, Bruno Pereira, Martin Soubrier, Jean-Jacques Dubost

**Affiliations:** 1 Department of Rheumatology, University Hospital Gabriel Montpied, 58 rue Montalembert, 63003 Clermont Ferrand CEDEX 1, France; 2 Biostatistics Unit, Clermont-Ferrand University Hospital, Clermont-Ferrand, France

**Keywords:** Infectious spondylodiscitis, Vertebral osteomyelitis, Coagulase-negative staphylococcus, *Staphylococcus aureus*

## Abstract

**Background:**

Coagulase-negative staphylococci (CoNS) are increasingly implicated in recent patient series of spondylodiscitis, but there are no series of CoNS-spondylodiscitis available. The objective of this study was to compare the characteristics of patients with spontaneous CoNS-spondylodiscitis with those patients with *Staphylococcus aureus* (SA) spondylodiscitis.

**Methods:**

This was a retrospective single center study involving 147 spontaneous infectious spondylodiscitis cases observed between 2000 and 2015. The 26 cases of CoNS-spondylodiscitis (15 confirmed) were compared with 30 cases of SA-spondylodiscitis. CoNS infection was considered confirmed if the same CoNS was isolated in at least two samples at two different times.

**Result:**

Patients with CoNS-spondylodiscitis were older (70 vs. 61 years of age; *p* = 0.01), had associated cancer more often (15% vs. 0%; *p* = 0.04) and had a longer diagnostic delay (>15 days in 88% vs. 60%; *p* = 0.01); experienced fever less often (19% vs. 50%; p = 0.01), and had lower white blood cell (7.6 vs. 9.9G/L; p = 0.01) and polymorphonuclear leucocyte counts (5.6 vs. 7.5G/L; *p* = 0.04). Patients with CoNS spondylodiscitis had less pronounced inflammatory syndrome (erythrocyte sedimentation rate [ESR]: 62 vs. 81 mm at 1 h; *p* = 0.03; CRP: 60 vs. 147 mg/L; *p* = 0.0003) and less common (ESR < 30 mm: 23% vs. 0%; *p* = 0.01; CRP < 10 mg/L: 23% vs. 0%; *p* = 0.005) in comparison with patients with SA infection. The infection entry site was most often an intravascular catheter (20% vs. 3%; *p* = 0.008). The level of positive percutaneous needle biopsies was comparable between CoNS and SA. Two patients who died both had SA infections.

**Conclusion:**

CoNS-spondylodiscitis involved at least 10% of spontaneous spondylodiscitis cases and was more common in elderly patients, afflicted by comorbidities, and its presentation was less virulent than that of those with SA-spondylodiscitis.

## Background

Infectious spondylodiscitis cases, like other osteoarticular infections, are mainly due to *Staphylococcus aureus* (SA). Coagulase-negative staphylococci (CoNS) have been implicated in patients with postoperative infectious spondylodiscitis. Isolated observations of spontaneous spondylodiscitis, primarily involving frail and immunosuppressed patients, are available in the literature [1, 2]. In the spontaneous spondylodiscitis series published, the frequency of CoNS ranged from 3% to 11% [3–8], with increasing involvement reported in the most recent series [3, 9–12]. CoNS infections pose a problem in terms of accountability: it is often difficult to confirm that it is responsible for the infection and not just present as contamination.

In order to further assess the frequency and characteristics of spontaneous CoNS-spondylodiscitis cases, we reviewed the medical files in the rheumatology department since 2000 of all patients who were hospitalized for infectious spondylodiscitis. The features of patients with CoNS-infections were compared to those with *S aureus* infections*.*

## Methods

This was a retrospective single center study focused on the medical files of patients hospitalized for infectious spondylodiscitis over the period from January 2000 to May 2015. The rheumatology department of Clermont-Ferrand University Hospital, France, is a tertiary regional care center. Our department admits patients suffering from osteoarticular as well as spine diseases. The patients are admitted directly, or via the emergency or other departments, though they may also be transferred from other hospitals. Postoperative and tuberculosis infections were excluded from the analysis. The diagnosis was considered confirmed when a radiologist with significant experience in osteoarticular pathology thought that the imaging examinations, particularly the magnetic resonance imaging, were suggestive of infectious spondylodiscitis. These results were considered, along with compatible clinical and laboratory presentations, in addition to the patient’s outcome after antibiotic therapy.

The data collected comprised demographic characteristics, infection site, microorganism type, identification method, initial clinical characteristics, risk factors including concomitant cancer, immunosuppressive treatment, ongoing corticoids therapy, diabetes, kidney insufficiency with renal clearance <30 mL/min or ongoing dialysis, drug addiction, severe liver disease, entry site (if identified), and laboratory parameters upon admission.

CoNS infection was considered confirmed if the same CoNS was isolated from two different samples (blood cultures, disco-vertebral biopsy, or another internal site) taken at different times. CoNS infection was considered probable if only one sample was positive.

Based on the data collected, comparisons were made between patients with CoNS-spondylodiscitis and those with SA-spondylodiscitis.

### Statistical analyses

Statistical analyses were performed using Stata software (version 13, StataCorp, College Station, TX.US). All statistical tests were two-tailed with a type I error at 5%. Continuous data were expressed as mean and 95% confidence interval (95% CI), depending on the statistical distribution, with an assumption of normality studied by the Shapiro-Wilk test. For quantitative data, the comparisons between groups (SA and CoNS, and confirmed and probable CoNS cases) were carried out using either Student’s t-test or Mann-Whitney test if the t-test conditions were not met ([i] normality and [ii] homoscedasticity studied by the Fisher-Snedecor test). For categorical parameters, the between-group comparisons were performed using Chi-squared test or Fisher’s exact test, as appropriate.

## Results

Between January 2000 and May 2015, a total of 147 patients were hospitalized in the rheumatology department for non-tuberculosis spontaneous infectious spondylodiscitis. Among these, 26 cases were due to CoNS, with the diagnosis confirmed in 15 and were considered probable in 11 cases (Table 1); 30 cases were due to SA. Overall, CoNS-spondylodiscitis cases involved 10% to 18% of the spontaneous infections according to the diagnostic criteria used. CoNS comprised *S. epidermidis* (*n* = 20), *S. simulans* (*n* = 1), *S. warneri* (n = 1), *S. lugdunensis* (n = 1),*S. hominis* (n = 1), *S. haemolyticus* (n = 1), and unclassified (n = 1).

**Table 1:** Bacteriological characteristics of patients with CoNS spondylodiscitis

Cases	Microorganism	Percutaneous needle biopsy			Blood cultures		Others	Probability
		Performed Y/N	Positive Y/N	Number of media	Number of blood cultures positive/ Number performed	Number of media		
1	*S. epidermidis*	Y	Y	1	0/10	0	N	probable
2	*S. epidermidis*	Y	Y	3	0/20	0	IV device	confirmed
3	*S.haemolyticus*	N	/	/	4/12	6	N	confirmed
4	*S. epidermidis*	Y	N	0	5/10	9	IV device	confirmed
5	*S. epidermidis*	Y	Y	2	2/14	2	N	confirmed
6	*S. epidermidis*	Y	Y	3	0/5	0	N	probable
7	*S. epidermidis*	Y	Y	3	0/9	0	N	probable
8	S.warneri	Y	Y	1	0/2	0	N	probable
9	*S. epidermidis*	Y ^a^	Y	3	0/16	0	N	confirmed
10	*S. epidermidis*	N	/	/	6/6	12	N	confirmed
11	*S. epidermidis*	Y	Y	2	4/14	7	IV device	confirmed
12	*S. epidermidis*	Y	Y	2	0/5	0	N	probable
13	*S.hominis*	Y	N	0	1/8	1	N	probable
14	*S.lugdunensis*	N	/	/	0/6	0	3 knee punctures	confirmed
15	S.simulans	N	/	/	3/3	3	N	confirmed
16	S. epidermidis	Y	Y	2	0/9	0	N	probable
17	S.coagulase negative	Y	Y	1	1/11	1	N	confirmed
18	*S. epidermidis*	Y	Y	3	5/5	6	N	confirmed
19	*S. epidermidis*	Y	Y	1	0/17	0	N	probable
20	*S. epidermidis*	N	/	/	6/8	11	N	confirmed
21	*S. epidermidis*	Y	Y	1	0/11	0	N	probable
22	*S. epidermidis*	Y	Y	1	0/6	0	N	probable
23	*S. epidermidis*	Y	Y	1	1/3	2	N	confirmed
24	*S. epidermidis*	N	/	/	2/10	3	N	confirmed
25	S. epidermidis	N	/	/	0/11	0	1 hip puncture	probable
26	*S.epidermidis*	Y	N	0	3/8	3	N	confirmed

Confirmed: CoNS were isolated from at least two different samples taken at different times. Probable: only one sample was positive

^a^Two percutaneous needle biopsy performed

### Comparison between CoNS-spondylodiscitis and SA-spondylodiscitis cases

The male predominance was similar for CoNS and SA infections (73 vs. 70%; p = NS) (Table 2). Patients with CoNS infection were older (70 vs. 61 years; *p* = 0.01) and had associated cancer more often (15 vs. 0%; *p* = 0.04). The frequency of diabetes in both was comparable. There were no differences in spondylodiscitis sites between the two groups. In patients infected with CoNS, there was a longer diagnostic delay than in those infected with SA (48 vs. 24 days; *p =* 0.004). This delay exceeded 15 days in 88% of patients with CoNS vs. 60% of patients with SA (*p* = 0.01). The CoNS infections were less often accompanied by fever (19% vs. 50%; *p* = 0.01), and with lower white blood cell (7.6 vs. 9.9G/L; *p* = 0.01) and polymorphonuclear leucocyte (5.6 vs. 7.5G/L; *p* = 0.04) counts. Inflammatory syndrome was less pronounced (erythrocyte sedimentation rate [ESR]: 62 vs. 81 mm at 1 h; *p* = 0.03; C-reactive protein [CRP]: 60 vs. 147 mg/L; *p* = 0.0003) and less common (ESR < 30 mm: 23% vs. 0%; p = 0.01; CRP < 10 mg/L: 23% vs. 0%; *p* = 0.005) compared to patients with SA infections. The entry site was an intravascular catheter more often (20 vs. 3%; *p* = 0.08). Blood cultures tended to be positive less often in those with CoNS infections (50% vs. 71%; *p* = 0.1), with percutaneous needle biopsies performed more often (73% vs. 37%; *p* = 0.008), yet with comparable positivity rates for CoNS and SA infection cases (84 vs. 82%; p = NS). Overall, CoNS infections in 10 patients (38%) and SA infections in six patients (20%) were methicillin-resistant (p = NS). The duration of the antibiotic therapy did not differ between the two groups, yet SA-spondylodiscitis cases had a longer hospital stay. It should be noted that the two patients who died were both infected with SA.

**Table 2:** Comparison of characteristics of patients with spondylodiscitis caused by coagulase-negative staphylococcus (CoNS) and caused by *Staphylococcus aureus*

	1: *S.aureus* *n* = 30	2: CoNS total *n* = 26	3: CoNS confirmed *n* = 15	4:CoNS probable *n* = 11	*p* 1 vs 2	*p* 1 vs 3	*p* 3 vs 4
Age (mean [95% CI])	61 [56; 67]	70 [66; 75]	71[64; 78]	70 [64; 76]	0.01	0.02	0.6
Male *n* (%)	21 (70)	19 (73)	11 (73)	8 (73)	0.8	0.8	1
Site *n* (%)					0.7	0.8	0.3
- Cervical	3 (10)	3 (12)	1 (7)	2 (18)			
- Thoracic	7 (23)	7 (27)	3 (20)	4 (36)			
- Lumbar	18 (60)	16 (62)	11 (73)	5 (45)			
Symptoms for more than 15 days *n* (%)	18 (60)	23 (88)	13 (87)	10 (91)	0.01	0.07	0.7
Duration (d) of hospitalization (median [95% CI])	28 [23.4;. 32.6]	21.5 [16.5; 26.4]	23.6 [15.8; 31.4]	18.5 [12.4; 24.7]	0.05	0.3	0.3
Temperature > 38 °C n (%)	15 (50)	5 (19)	5 (33)	0 (0)	0.01	0.3	0.05
Presence of risk factors n (%)	14 (47)	15 (58)	9 (60)	6 (55)	0.4	0.4	0.8
Cancer *n* (%)	0 (0)	4 (15)	3(20)	1 (9)	0.04	0.03	0.6
Diabetes *n* (%)	9 (30)	8 (31)	5 (33)	3 (27)	0.9	0.8	1
Entry site							
-Cutaneous n (%)	9 (30)	6 (23)	2 (13)	4 (36)	0.5	0.3	0.13
-Catheter *n* (%)	1 (3)	5 (19)	3 (20)	2(18)	0.08	0.1	1
Blood cultures n (%)	20 (71)	13 (50)	12 (80)	1 (9)	0.1	0.5	0.001
Percutaneous needle biopsy made n (%)	11 (37)	19 (73)	9 (60)	10 (90)	0.008	0.1	0.07
Positive percutaneous needle biopsy n (%)	9 (82)	16 (84)	7 (78)	9 (90)	0.8	0.8	0.46
Leukocytes (G/L) (mean [95% CI])	9.9 [8.4; 11.4]	7.6 [6.6; 8.6]	8.3 [6.8; 9.7]	6.7 [5.2; 8.3]	0.01	0.5	0.1
PMN (G/L) (mean [95% CI])	7.5 [6.0; 9.0]	5.6 [4.6; 6.6]	6.3 [4.9; 7.7]	4.6 [3.2; 6.0]	0.04	0.1	0.07
ESR < 30 at 1 h *n* (%)	0 (0)	5 (23)	2(15)	3(33)	0.01	0.1	0.3
CRP > 10 mg/L *n* (%)	30 (100)	20 (77)	12(80)	8(73)	0.005	0.02	0.6
Antibiotic therapy duration (d) (median [95% CI])	91.5 [84.4; 98.7]	82.6 [74.5; 90.6]	75.6 [63.7; 87.5]	93.0 [86.2; 99.8]	0.1	0.05	0.09
Sensitivity to methicillin *n* (%)	24(80)	16 (61)	10(67)	6(55)	0.1	0.2	0.7

*PMN* polymorphonuclear leucocytes, *ESR* erythrocyte sedimentation rate, *CRP* C-reactive protein

### Comparison of confirmed CoNS-spondylodiscitis and SA-spondylodiscitis cases

Patients with confirmed CoNS infections were older than those with SA infections (Table 2) and had associated cancer more often (20% vs. 0%; *p* = 0.04). Additionally, inflammatory syndrome was less pronounced (CRP: 76 vs. 147 mg/L; *p* = 0.02), with CRP <10 mg/L in 20% of CoNS infection cases versus CRP >10 mg/L in all SA infection cases. Antibiotic therapy duration was shorter in patients with confirmed CoNS spondylodiscitis versus those with SA spondylodiscitis (75 vs. 91 days; *p* = 0.05).

## Discussion

In our cohort of spontaneous spondylodiscitis cases, at least 10% were caused by CoNS infections. Compared to SA spondylodiscitis cases, CoNS spondylodiscitis affected older patients, with concomitant cancer and who exhibited less virulent infection symptoms. One out of 5 entry sites was an intravascular catheter.

Over recent years, CoNS infections have been shown to be increasing in frequency [13]. Most often, these infections are of nosocomial origin and are associated with foreign object implantations, mostly prostheses and catheters [14]. The main difficulty consists of confirming the microorganism responsible, given that there is no reliable tool to differentiate between infection and contamination, though most authors deem infection to be highly likely when the same microorganism is isolated in at least two samplings taken at two different times [13, 15].

The frequency of CoNS spondylodiscitis was shown to vary greatly among the series published in the literature, depending mostly on the inclusion criteria. The frequency rates vary from 3% to 11% in spontaneous spondylodiscitis series [3–8]. In a recent French multicenter study on spontaneous spondylodiscitis with negative blood cultures, CoNS was the microorganism most frequently identified by percutaneous needle biopsy, representing 18% of the spondylodiscitis (17/ 95) and 61% (17/28) of the staphylococcal spondylodiscitis cases [16].This microorganism has been increasingly implicated in the most recent series, and Lora Tamayo et al. suggested that the higher frequency of these slightly virulent microorganism translates may be accounted for by epidemiological changes [3]. This may result from the increased age of the populations and comorbidities, particularly cancer, as seen in our study. Moreover, because catheters are a common portal entry for CoNS, hemodialysis has not surprisingly been reported as a relevant risk factor for CoNS spondylodiscitis [17]. The increase in the occurrence of CoNS spondylodiscitis likely reflects the increased infection rates caused by this microorganism, as noted over recent years [8].

CoNS infection is indolent and is characterized by largely non-specific symptoms and an attenuated infectious syndrome. Less than one-third of patients display fever. In one out of five cases in our study infectious syndrome was completely lacking accountings for the substantial diagnostic delay. The indolence appeared to be even more pronounced in patients with probable CoNS, although we cannot exclude contamination in some cases. The low frequency of positive blood cultures retrieved from this group can probably be explained, at least to some extent, by the inclusion criteria used.

Common resistance to antibiotics proves to be another characteristic of CoNS infection. In our cohort, a higher level of methicillin-resistant strains was found in CoNS cases, compared to SA cases, but the difference did not reach statistical significance, probably because the low patient numbers. The frequency of methicillin-resistant strains identified in our cohort was comparable to that revealed in osteoarticular infections in a French orthopedic department, with 23% of SA and 44% of CoNS cases found to be resistant to methicillin [18].

This study has several limitations, particularly its retrospective study design. In addition, because of its center design, extrapolation of our results to other centers requires great caution. Though this cohort is the largest of its kind, the number of patients with confirmed CoNS spondylodiscitis was relatively small, which may have limited the study’s potential to detect differences with the SA spondylodiscitis group.

## Conclusion

While waiting for reliable bacteriological techniques capable of differentiating between infection and contamination, identifying CoNS from a bacteriological examination must be considered for the diagnosis of spondylodiscitis, and a systematic conclusion on contamination should be avoided.

## Abbreviations

CoNS: Coagulase-negative staphylococcal
CRP: C-reactive protein
ESR: Erythrocyte sedimentation rate
MRI: Magnetic resonance imaging
PMN: Polymorphonuclear leucocytes
SA: *Staphylococcus aureus*
